# G Protein βγ-Subunit Signaling Mediates Airway Hyperresponsiveness and Inflammation in Allergic Asthma

**DOI:** 10.1371/journal.pone.0032078

**Published:** 2012-02-22

**Authors:** Gustavo Nino, Aihua Hu, Judith S. Grunstein, Joseph McDonough, Portia A. Kreiger, Maureen B. Josephson, John K. Choi, Michael M. Grunstein

**Affiliations:** 1 Division of Pulmonary Medicine, Children's Hospital of Philadelphia Research Institute, University of Pennsylvania School of Medicine, Philadelphia, Pennsylvania, United States of America; 2 Division of Pediatric Pulmonary and Sleep Medicine, Pennsylvania State University College of Medicine, Hershey, Pennsylvania, United States of America; 3 Department of Pathology and Laboratory Medicine, Children's Hospital of Philadelphia Research Institute, University of Pennsylvania School of Medicine, Philadelphia, Pennsylvania, United States of America; 4 Department of Pathology, Nemours/A.I. duPont Hospital for Children, Wilmington, Delaware, United States of America; University of Pecs Medical School, Hungary

## Abstract

Since the Gβγ subunit of Gi protein has been importantly implicated in regulating immune and inflammatory responses, this study investigated the potential role and mechanism of action of Gβγ signaling in regulating the induction of airway hyperresponsiveness (AHR) in a rabbit model of allergic asthma. Relative to non-sensitized animals, OVA-sensitized rabbits challenged with inhaled OVA exhibited AHR, lung inflammation, elevated BAL levels of IL-13, and increased airway phosphodiesterase-4 (PDE4) activity. These proasthmatic responses were suppressed by pretreatment with an inhaled membrane-permeable anti-Gβγ blocking peptide, similar to the suppressive effect of glucocorticoid pretreatment. Extended mechanistic studies demonstrated that: 1) corresponding proasthmatic changes in contractility exhibited in isolated airway smooth muscle (ASM) sensitized with serum from OVA-sensitized+challenged rabbits or IL-13 were also Gβγ-dependent and mediated by MAPK-upregulated PDE4 activity; and 2) the latter was attributed to Gβγ-induced direct stimulation of the non-receptor tyrosine kinase, c-Src, resulting in downstream activation of ERK1/2 and its consequent transcriptional upregulation of PDE4. Collectively, these data are the first to identify that a mechanism involving Gβγ-induced direct activation of c-Src, leading to ERK1/2-mediated upregulation of PDE4 activity, plays a decisive role in regulating the induction of AHR and inflammation in a rabbit model of allergic airway disease.

## Introduction

G proteins play critical roles in regulating the allergic asthmatic phenotype, including the induction of airway hyperresponsiveness (AHR) and inflammation [1]. The G proteins are heterotrimers comprised of α, β and γ subunits and, upon activation by G protein-coupled receptors (GPCRs) that respond to a host of bronchoactive and proinflammatory stimuli, the Gα subunit undergoes an exchange of GTP for GDP and becomes dissociated from the Gβγ subunits [2]. Both the free Gα and Gβγ subunits can then activate different effectors, importantly including those stimulating the MAPK signaling pathways that regulate various immune and inflammatory cell functions [3]. The MAPK pathways are also implicated in regulating different aspects of airway smooth muscle (ASM) function and inflammation under proasthmatic conditions, including activation of transcription factors and other downstream effectors that mediate the release of proinflammatory cytokines and chemokines which can alter ASM contractility and growth [4]–[6].

Previous studies demonstrated that the class of pertussis toxin (PTX)-sensitive G proteins that inhibit adenylate cyclase activity (i.e., Gi proteins) plays a particularly important role in mediating the heightened agonist-induced constrictor responses and impaired β2-adrenoceptor (β2AR)-induced relaxation responses exhibited in isolated ASM tissues exposed to different proasthmatic conditions, including passive sensitization with serum from atopic asthmatic patients [7], proinflammatory cytokine exposure [8], and inoculation with rhinovirus [9]. More recently, we reported that PTX-sensitive proasthmatic changes in ASM responsiveness are also exhibited in ASM tissues following their prolonged heterologous or homologous β2AR desensitization, and that this altered ASM function is attributed to upregulated phosphodiesterase 4 (PDE4) activity induced by activation of the Gβγ subunit of Gi protein [10], [11]. Specifically, Gβγ signaling was found to activate the non-receptor tyrosine kinase, c-Src, which stimulates the Ras/c-Raf1/MEK signaling pathway leading to downstream activation of the MAPK, ERK1/2, the latter evoking transcriptional upregulation of PDE4 activity [10], [11]. Collectively, these findings were consistent with the prevailing concept that GPCR-dependent and receptor-independent stimulation of Ras-mediated ERK1/2 activation uses proximal signals generated by the βγ subunits of G protein coupled to c-Src activation [12]–[16]. In light of this evidence, together with that implicating an important causal relationship between PTX-sensitive Gβγ signaling and inflammation [17]–[22], the present study addressed the hypothesis that Gβγ signaling regulates the altered airway responsiveness and inflammation exhibited in the allergic asthmatic state. The results obtained in studies conducted in an *in vivo* rabbit model of allergic asthma and in isolated atopic sensitized ASM tissues are the first to demonstrate that: 1) inhibition of Gβγ signaling prevents the induction of airway hyperresponsiveness and inflammation elicited by antigen challenge in allergic rabbits, as well as the pro-asthmatic changes in constrictor and relaxation responsiveness exhibited in atopic sensitized ASM tissues; and 2) these bronchoprotective actions of Gβγ inhibition are attributed to suppression of Gβγ-induced direct activation of c-Src, which leads to downstream ERK1/2-dependent upregulation of PDE4 activity and its consequent pro-asthmatic effects on airway function. Taken together, these new findings highlight a heretofore-unidentified pivotal role for Gβγ signaling in regulating the airway asthmatic phenotype, and suggest that interventions targeted at suppressing Gβγ signaling associated with Gi protein activation may lead to new approaches to treat allergic airway disease.

## Results

### Gβγ-coupled ERK1/2 and PDE4 activation mediates altered constrictor and relaxation responsiveness in atopic sensitized ASM

**Figure 1:**
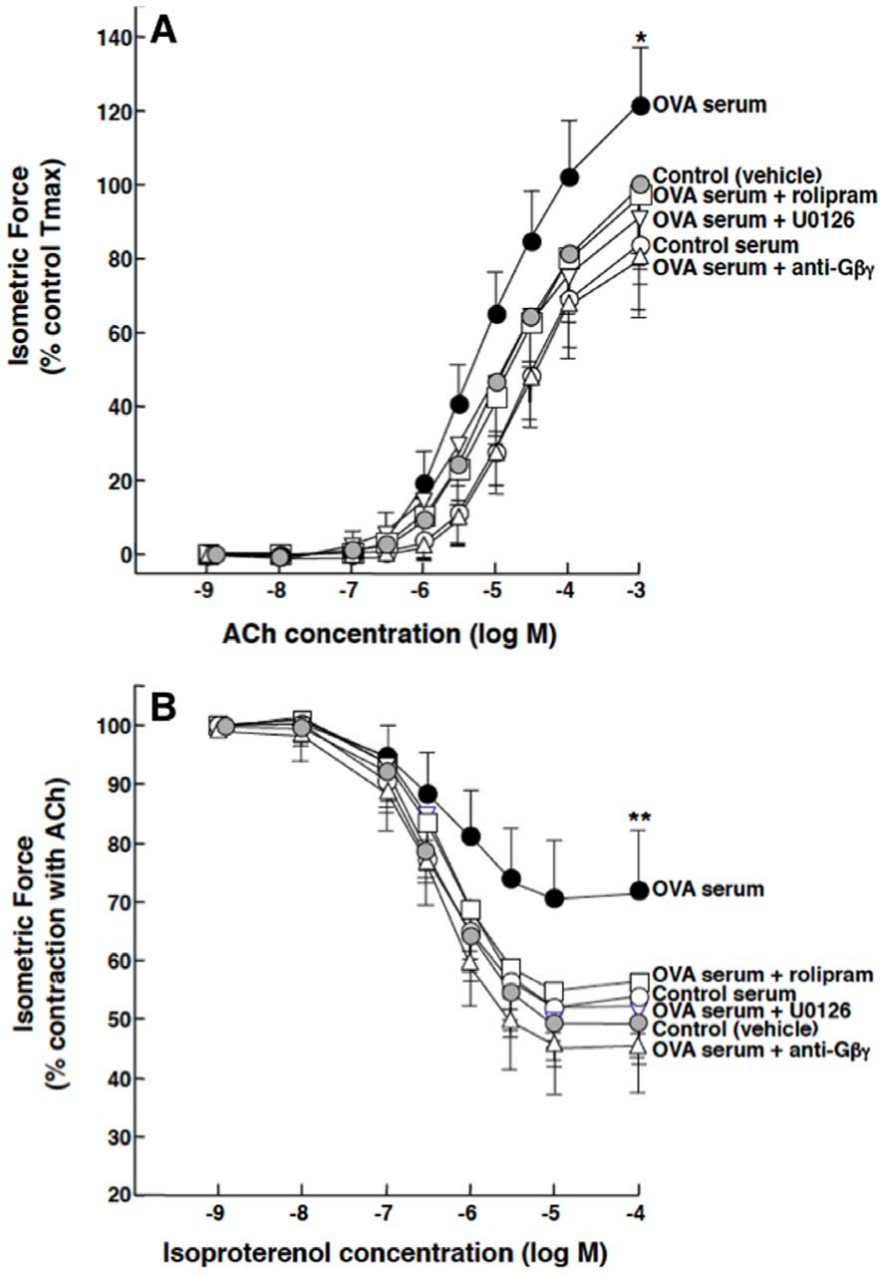
Inhibition of ERK1/2, PDE4, or Gβγ signaling prevents induced changes in agonist responsiveness in OVA-serum-sensitized ASM tissues. Relative to vehicle- or control serum-exposed rabbit ASM tissues, tissues passively sensitized for ∼18 hr with OVA serum exhibit significantly increased contractility to ACh (**A**) and impaired relaxation to isoproterenol (**B**). Pre-treatment with either U0126, rolipram, or anti-Gβγ peptide prevents OVA serum-induced changes in ASM responsiveness. Data are mean ± SD values from 5–7 experiments. ANOVA used for multiple comparisons of mean Tmax values. *p<0.05; **p<0.01.

Given recent evidence demonstrating that transcriptional upregulation of PDE4 activity due to Gi-βγ-regulated activation of ERK1/2 mediates proasthmatic changes in agonist responsiveness in β2AR-desensitized ASM [10], [11], [23], we initially examined whether these signaling molecules also participate in mediating the reported IgE-induced Gi protein-dependent proasthmatic changes in responsiveness exhibited in ASM tissues passively sensitized with atopic asthmatic serum [7], [24]. Accordingly, agonist-induced constrictor and relaxation responses were compared in isolated naïve rabbit ASM tissues that were incubated overnight with vehicle alone (control) or serum isolated from either non-sensitized (control serum) or OVA-sensitized rabbits at 24 hr following OVA inhalation (OVA serum), both in the absence and presence of pretreatment with either the PDE4 inhibitor, rolipram (10 µM), the ERK1/2 inhibitor, U0126 (5 µM), or a Gβγ sequestering (blocking) peptide (20 µM) comprised of the C-terminal domain of phosducin-like protein (PhLP) conjugated to an inert membrane permeable peptide (MPS) carrier [25]. The latter anti-Gβγ blocking peptide was previously shown to inhibit Gi protein-dependent upregulation of PDE4 activity and its consequent induction of altered responsiveness in β2AR-desensitized ASM [11]. As shown in Fig. 1A, relative to the similar responses obtained in vehicle- and control serum-exposed tissues, OVA serum-sensitized ASM tissues exhibited significantly increased constrictor responsiveness to cumulative administration of ACh, yielding a mean ± SD maximal constrictor response (Tmax) that averaged 121.7±16.7% of control (p<0.05). This enhanced contractility was completely abrogated in OVA serum-sensitized tissues that were pretreated either with the anti-Gβγ blocking peptide, rolipram, or U0126, with no significant differences observed between the protective effects of these inhibitors.

Under the same treatment conditions, cumulative administration of the β2AR agonist, isoproterenol, produced dose-dependent relaxation of half-maximally pre-constricted ASM segments (Fig. 1B). Relative to controls, the relaxation responses were significantly attenuated in the OVA serum-sensitized tissues, wherein the mean ± SE maximal relaxation response (Rmax) amounted to 27.8±12.1% vs. 46.1±11.1% in the controls (p<0.01). This impaired relaxant responsiveness was also ablated in OVA serum-exposed tissues that were pretreated either with the anti-Gβγ blocking peptide, rolipram, or U0126, with no significant differences detected between the protective effects of these inhibitors. Of note, results obtained in related experiments demonstrated that: 1) relative to untreated (vehicle-exposed) tissues, neither the Tmax nor Rmax responses to ACh and isoproterenol, respectively, were significantly affected in control serum-exposed ASM tissues that were pretreated either with the inert MPS carrier peptide alone or anti-Gβγ blocking peptide; and 2) in contrast to the protective effects of the anti-Gβγ peptide in OVA serum-sensitized ASM tissues, pretreatment of these tissues with MPS alone did not significantly affect their altered agonist responsiveness (Fig. S1). Moreover, as we previously reported in naïve rabbit ASM tissues [10], [11], [23], neither co-incubation with rolipram, U0126, nor the anti-Gβγ peptide significantly affected the responsiveness of control serum-exposed ASM tissues to either ACh or isoproterenol (data not shown). Thus, in demonstrating that inhibition of either Gβγ, ERK1/2 or PDE4 signaling prevents the changes in agonist responsiveness elicited in OVA serum-sensitized ASM, these observations are consistent with those previously reported in β2AR-desensitized ASM wherein activation of the Gβγ subunit of Gi protein was also found to initiate ERK1/2-dependent rolipram-sensitive proasthmatic changes in constrictor and relaxation responsiveness [10], [11], [23].

### Gβγ signaling mediates *in vivo* airway hyperresponsiveness in allergic rabbits

**Figure 2:**
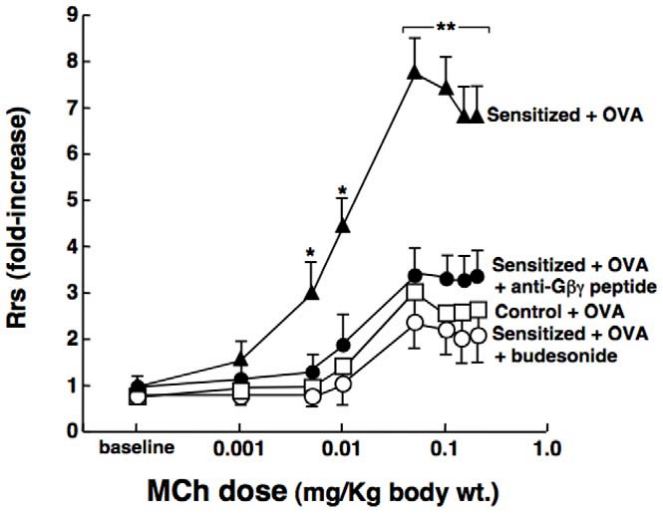
Anti-Gβγ blocking peptide prevents *in vivo* antigen-induced airway hyperresponsiveness in OVA-sensitized rabbits. Relative to OVA-challenged control (non-sensitized; n = 4) rabbits, Rrs responses to MCh are significantly increased at 24 hr following antigen challenge in OVA-sensitized rabbits (n = 4). This heightened bronchoconstrictor responsiveness to MCh is suppressed in OVA-sensitized rabbits that are treated either with inhaled anti-Gβγ peptide (1 mg/Kg; n- = 4) or budesonide (0.5 mg/Kg; n = 3) prior to antigen challenge. Data are mean ± SE values. ANOVA used for multiple comparisons of mean Rrs values. *p<0.05; **p<0.01. Note: a significant difference is also detected when using the nonparametric Kruskal-Wallis test with Dunn's post-test to compare the medians of the Rrs-max responses in the control vs. non-pretreated OVA sensitized rabbits (p<0.05), whereas no significant difference is detected between the control vs. OVA-sensitized animals that are pretreated with the anti-Gβγ blocking peptide.

To determine whether Gβγ inhibition exerts a comparable bronchoprotective action *in vivo*, we examined the effect of pretreatment with aerosolized anti-Gβγ blocking peptide on bronchoconstrictor responsiveness to MCh in OVA-sensitized rabbits, and compared this effect to that of the inhaled glucocorticosteroid, budesonide. The measurements of baseline respiratory system resistance (Rrs) obtained at 24 hr following antigen challenge in non-sensitized (control) and OVA-sensitized rabbits were not significantly different, averaging 0.029±0.004 and 0.031±0.005 cmH20/ml/sec, respectively. Relative to controls, however, the OVA-sensitized rabbits exhibited pronounced AHR to i.v. administration of MCh, as evidenced by markedly heightened dose-dependent increases in Rrs (Fig. 2). The mean ± SE maximal MCh-induced increase in Rrs (Rrs-max) averaged 7.81±0.74-fold above baseline in the OVA-sensitized rabbits vs. an increase of 3.01±0.60-fold in the control animals (p<0.001). This AHR was virtually completely suppressed in OVA-sensitized rabbits that were treated with inhaled anti-Gβγ blocking peptide prior to OVA challenge, similar to the suppression of AHR exhibited in OVA-sensitized rabbits that were pretreated with inhaled budesonide (Fig. 2). Of note, comparable significant differences were also detected when analyzing these data using the nonparametric Kruskal-Wallis test (p = 0.023), with the Dunn's post-test demonstrating a significant difference (p<0.05) when comparing the median Rrs-max value in the control (2.69; range: 2.10–4.89) vs. non-pretreated OVA sensitized rabbits (7.29; range: 6.05–9.84), whereas no significant difference was detected when comparing the median of the controls vs. median Rrs-max of 3.22 (range: 6.95–9.84) obtained in the OVA-sensitized animals that were pretreated with the anti-Gβγ blocking peptide. In relation to these observations, it should be noted that results generated in separate experiments demonstrated that, relative to the above corresponding determinations: 1) bronchoconstrictor responsiveness to MCh was unaffected in control rabbits that were treated with anti-Gβγ peptide (i.e., Rrs-max = 2.82±0.63-fold above baseline; n = 3); 2) the AHR evoked by antigen challenge was not significantly altered in OVA-sensitized rabbits that were treated with MPS alone (i.e., Rrs-max = 6.95±0.84-fold above baseline; n = 3); and 3) suppression of AHR comparable to that observed in anti-Gβγ blocking peptide- or budesonide-treated rabbits was also detected in OVA-sensitized rabbits that were pretreated with gallein (30 mg/Kg; n = 4), a small molecule inhibitor of Gβγ signaling [17], , yielding Rrs-max values of 2.54±0.72-fold above baseline. Finally, in accordance with the above observations, comparable results were also obtained when analyzing MCh responsiveness in terms of the corresponding changes in dynamic compliance (Cdyn) measured under the different experimental conditions. The baseline Cdyn values were lower in the OVA-sensitized+challenged vs. control rabbits, averaging 3.71±0.06 vs. 4.45±0.04 ml/cmH20, respectively (p<0.05) and, as depicted in Fig. S2: 1) relative to controls, MCh-induced decreases in Cdyn (expressed as % of baseline) were significantly greater in the OVA-sensitized+challenged rabbits; and 2) this induced change in the Cdyn responses to MCh was suppressed in OVA-sensitized rabbits that were treated with anti-Gβγ blocking peptide before antigen challenge, similar to the suppression exhibited in OVA-sensitized rabbits that were pretreated with budesonide. As with the above Rrs data, comparable significant differences were also detected when using the nonparametric Kruskal-Wallis test and Dunn's post-test to compare the Cdyn responses generated in the control vs. OVA-sensitized rabbits in the absence (p<0.05) vs. presence (p = NS) of pretreatment with anti-Gβγ blocking peptide.

### Gβγ signaling mediates allergic airway inflammation in OVA-sensitized rabbits

**Figure 3:**
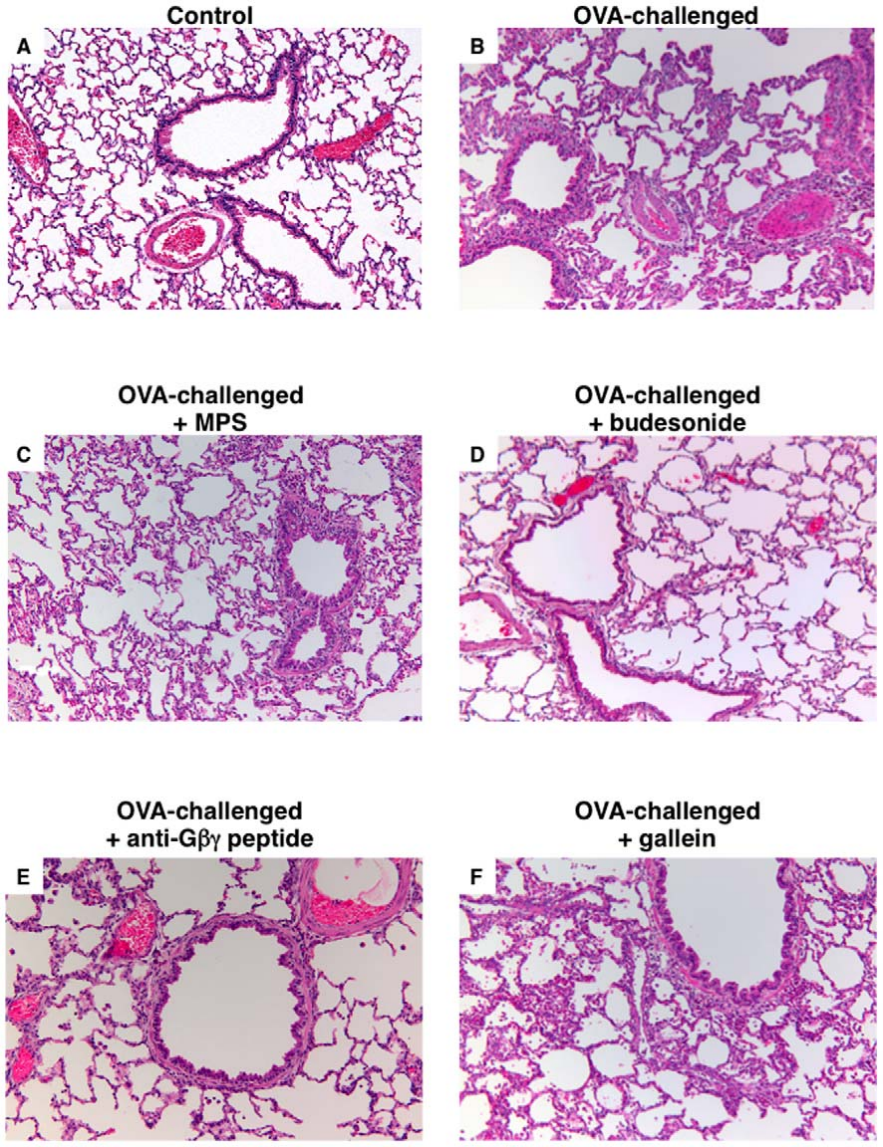
Anti-Gβγ blocking peptide suppresses pulmonary inflammation in OVA-sensitized rabbits. Relative to controls (**A**), lungs isolated from antigen-challenged OVA-sensitized rabbits exhibit diffusely scattered patchy foci of inflammatory cell infiltration, including in peribronchial, perivascular, and parenchymal regions (**B**). Contrasting the lack of effect of pretreatment with MPS alone (**C**), inflammation is suppressed to a similar extent in antigen-challenged OVA-sensitized rabbits that were pretreated either with inhaled budesonide (**D**) or anti-Gβγ peptide (**E**), whereas pretreatment with gallein has relatively little anti-inflammatory effect (**F**). Representative photomicrographs (mag. ×100) are from 4 µM sections of H&E stained lung sections.

**Figure 4:**
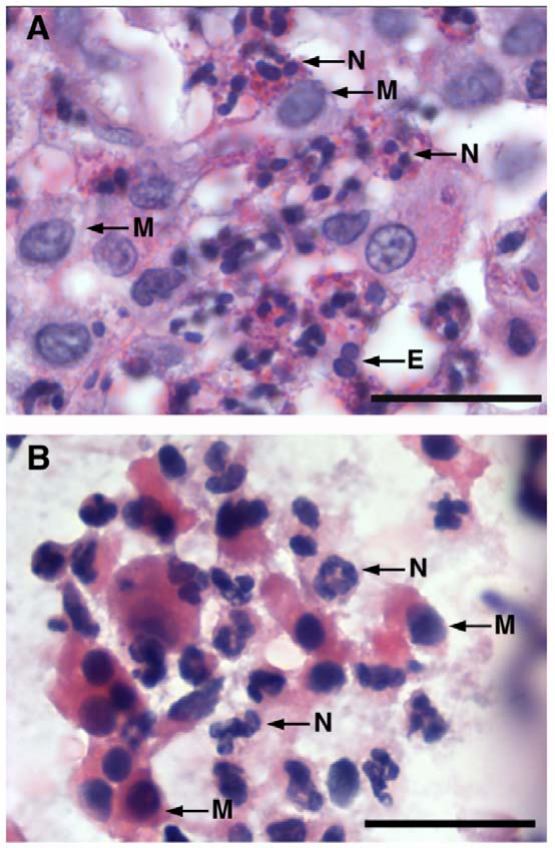
Pulmonary inflammatory response to antigen challenge in OVA-sensitized rabbits. Representative high magnification photomicrographs (mag. ×1000) demonstrating that inflammatory infiltrate in lung tissues (**A**) and BALF (**B**) isolated from OVA-sensitized+challenged rabbits is mostly composed of neutrophils (N) with lesser amounts of mononuclear macrophages (M) and rare eosinophils (E).

Together with its above bronchoprotective action, inhaled pretreatment with anti-Gβγ blocking peptide also suppressed the pulmonary inflammatory response detected in the sensitized rabbits at 24 hr following OVA challenge. As shown by the representative lung sections in Fig. 3, relative to control (non-sensitized) rabbits that showed no sign of inflammation (Fig. 3A), lungs isolated from OVA-sensitized+challenged rabbits exhibited multiple patchy foci of peribronchial, perivascular, and parenchymal inflammation (Fig. 3B). The inflammatory cell infiltrates consisted mainly of neutrophils and, to a lesser extent, macrophages and rare eosinophils, as depicted in the high magnification photomicrographs of representative lung tissue and BAL fluid (BALF) samples in Figs. 4A and 4B, respectively. In contrast to the lack of effect of pretreatment with MPS alone (Fig. 3C), the inflammatory response was distinctly suppressed to a similar extent in OVA-sensitized+challenged rabbits that were pretreated with either inhaled budesonide or anti-Gβγ blocking peptide (Figs. 3D and 3E, respectively). Interestingly, by comparison, the inflammatory response was largely unaffected in OVA-sensitized+challenged rabbits that were pretreated with gallein (Fig. 3F), suggesting differences between this small molecule inhibitor of Gβγ and the anti-Gβγ blocking peptide with respect to their anti-inflammatory actions (see Discussion).

**Figure 5:**
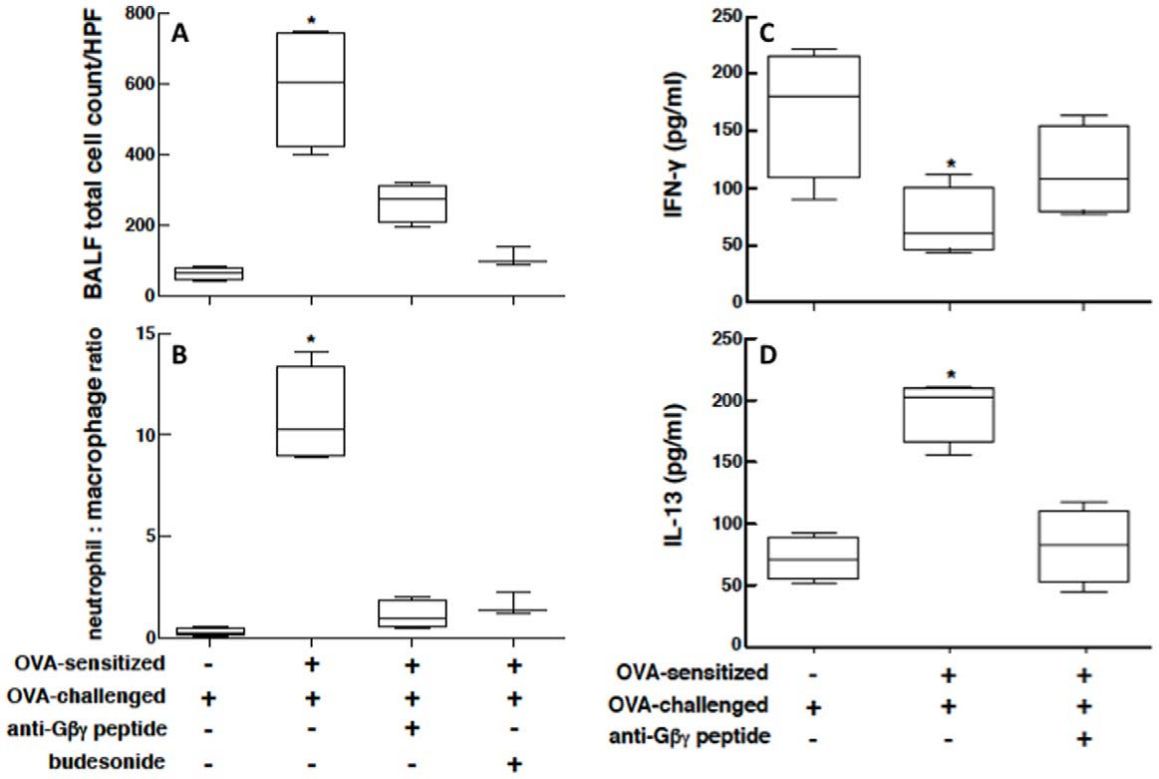
Proinflammatory cellular and cytokine responses in antigen-challenged OVA-sensitized rabbits are suppressed by anti-Gβγ blocking peptide. Relative to controls, total inflammatory cell count (**A**) and neutrophil/macrophage cellular ratio (**B**), are significantly increased in BALF samples from antigen-challenged OVA-sensitized rabbits. Correspondingly, BALF levels of INF-γ are significantly reduced whereas IL-13 levels are increased in OVA-sensitized+challenged animals. These proinflammatory indices are suppressed in BALF from sensitized rabbits that are treated with inhaled anti-Gβγ peptide prior to OVA challenge. Data are represented by bar plots depicting median and range values. Statistical comparisons are made using the Kruskal-Wallis test. *p<0.05 based on Dunn's post-test.

Comparable results were obtained with respect to the corresponding changes in BALF cellular content and cytokine levels. Due to variability in recovery and dilution between the individual BALF samples, these data were analyzed with respect to changes in inflammatory cell counts per high power field (HPF) in BALF cytospins (Fig. 5A), wherein at least 500 cells were counted in each cytospin preparation, and as changes in the neutrophil/macrophage cell ratio (Fig. 5B). The box plots in Fig. 5 depict the median and range of the values determined under each experimental condition, and the nonparametric Kruskal-Wallis test with Dunn's post-test analysis demonstrated that, as compared to non-sensitized (control) rabbits challenged with OVA, the OVA-sensitized+challenged animals exhibited significant increases (p<0.05) in both the total number of cells (Fig. 5A) and neutrophil/macrophage cell ratio (Fig. 5B). Moreover, after antigen challenge, the control vs. OVA-sensitized rabbits also exhibited differences in their BALF levels of the signature Th1- and Th2-type cytokines, IFN-γ and IL-13, respectively. Compared to controls, the levels of IFN-γ were significantly reduced (p<0.05) in the OVA-sensitized+challenged rabbits (Fig. 5C), whereas the IL-13 levels (Fig. 5D) were significantly increased (p<0.05). As further depicted, the induced changes in the above inflammatory indices were suppressed in OVA-sensitized+challenged rabbits that were pretreated with inhaled anti-Gβγ blocking peptide, with no sigificant differences detected when compared to the control animals.

### Gβγ activation regulates PDE activity in OVA-challenged allergic lungs and sensitized ASM tissues

**Figure 6:**
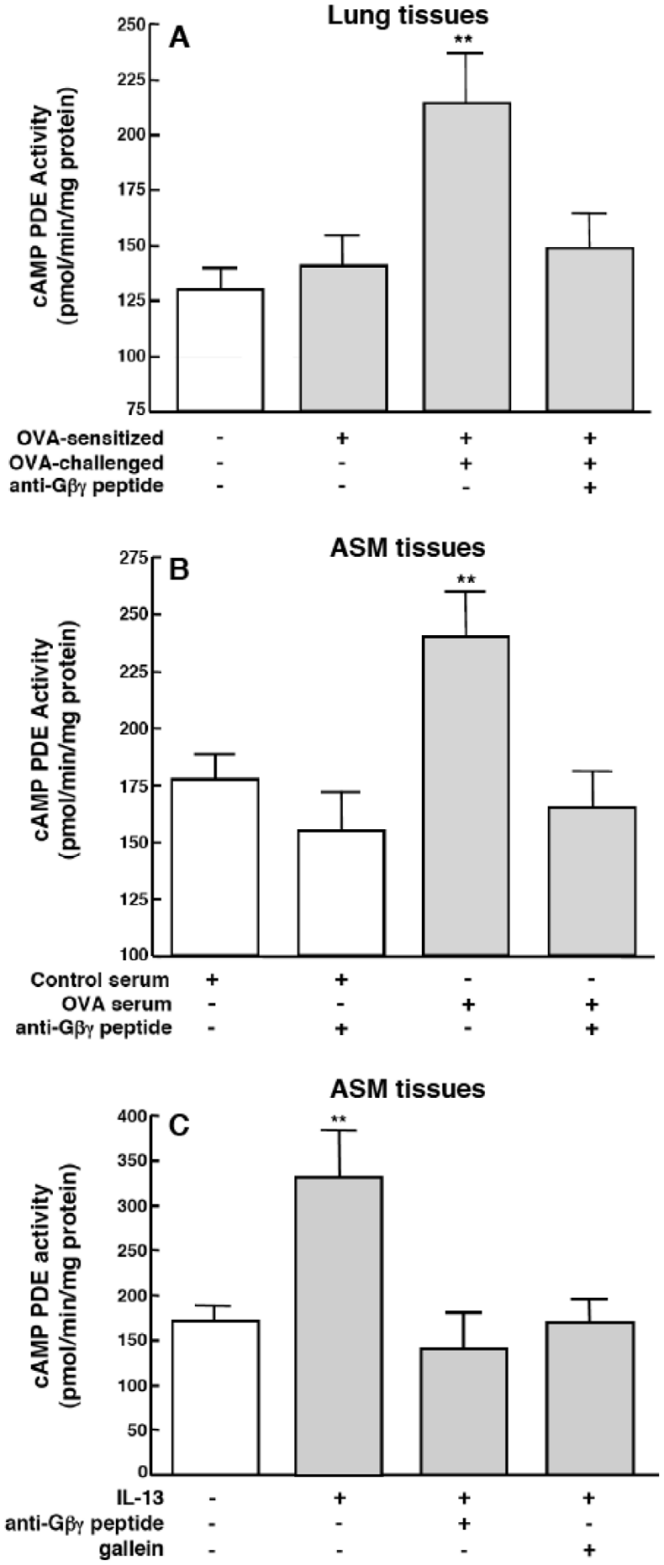
Inhibition of Gβγ-signaling prevents upregulation of PDE activity in lungs from OVA-sensitized/challenged rabbits and passively sensitized ASM tissues. Relative to controls, PDE activity is significantly increased in lung tissues isolated from OVA-sensitized+challenged rabbits (**A**) and in OVA serum-sensitized (**B**) and IL-13-treated (**C**) isolated ASM tissues. The upregulated PDE activity is abrogated in lungs isolated from OVA-sensitized rabbits that are treated with inhaled anti-Gβγ peptide prior to antigen challenge, as well as in OVA serum- or IL-13-exposed ASM tissues that were pretreated with anti-Gβγ peptide or gallein. Data are mean ± SE values from 3–5 determinations. Comparisons are made using two-tailed Student t-test. **p<0.01.

Upregulated PDE4 activity was shown to play a determinant role in mediating airway hyperresponsiveness and inflammation in response to allergen challenge in asthmatic individuals [28] and in animal models of allergic asthma [29]–[33]. Given this evidence, together with the above data implicating an association between Gβγ activation and rolipram-sensitive proasthmatic changes in responsiveness in OVA serum-sensitized ASM tissues (Fig. 1), we next examined whether lung tissues isolated from OVA-sensitized+challenged rabbits and ASM tissues passively sensitized with sera from these animals exhibit Gβγ-dependent changes in cAMP PDE activity. Relative to peripheral lung tissue sections isolated from control rabbits, as well as sensitized rabbits that were not challenged with OVA, significantly increased levels of PDE activity were detected in the lung tissues of OVA-sensitized+challenged rabbits (Fig. 6A). Similarly, relative to naïve ASM tissues exposed to control serum, significantly increased levels of PDE activity were detected in ASM tissues that were passively sensitized with OVA serum (Fig. 6B). This upregulated PDE activity was abrogated in lungs from OVA-sensitized+challenged rabbits that were pretreated *in vivo* with anti-Gβγ blocking peptide, and the latter pretreatment also suppressed the increased PDE activity in OVA serum-exposed ASM tissues (Figs. 6A and 6B, respectively). Of note, comparable results were also obtained in separate experiments wherein naïve ASM tissues were exposed for 24 hr to a maximally effective concentration of IL-13 (50 ng/ml) [34], the Th2 cytokine that is critically implicated in mediating antigen-induced airway hyperresponsiveness *in vivo* [35], [36], as well as the pro-asthmatic changes in contractility in atopic serum-sensitized ASM tissues, the latter attributed to IgE-induced activation of its low affinity receptor (CD23) [24], [34]. As shown in Fig. 6C, relative to controls, IL-13-treated ASM tissues exhibited significantly increased PDE activity that was suppressed by pretreatment with either the anti-Gβγ blocking peptide or gallein (10 µM).

### Gβγ regulates c-Src-coupled ERK1/2 activation in IL-13-exposed human ASM cells

**Figure 7:**
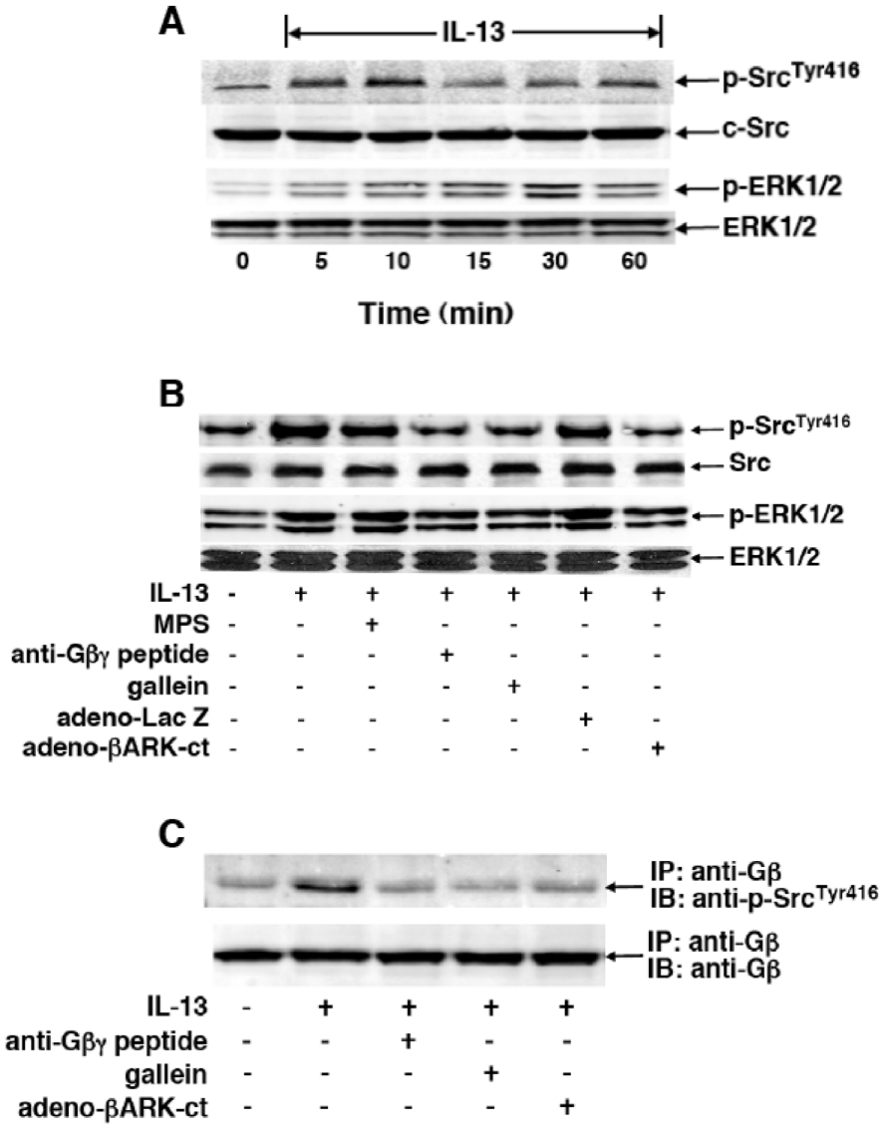
Gβγ signaling regulates IL-13-induced c-Src and ERK1/2 activation in HASM cells. (**A**) Immunoblots depicting IL-13-induced transient phosphorylation of c-Src^Tyr416^ and ERK1/2 in HASM cells, with peak phosphorylation detected at 10 and 30 min, respectively. (**B**) Immunoblots depicting that, contrasting the lack of effect of pretreatment with MPS alone, IL-13-induced phosphorylation of c-Src^Tyr416^ and ERK1/2 is suppressed in HASM cells pretreated with either anti-Gβγ peptide (20 µM) or gallein (10 µM). Additionally, in contrast to HASM cells transfected with adeno-LacZ (i.e., negative control), IL-13-induced phosphorylation of c-Src^Tyr416^ and ERK1/2 is also suppressed in HASM cells wherein Gβγ signaling is inhibited by transfection with adeno-βARK-ct. (**C**) Western blot depicting interaction of Gβ and c-Src^Tyr416^ in HASM cells stimulated with IL-13 in the absence and presence of inhibition of Gβγ activation. Following preparation of lysates from untreated and IL-13-treated (50 ng/ml×10 min) HASM cells, Gβ was immunoprecipitated (IP) with anti-Gβ monoclonal antibody, and subsequently immunoblotted (IB) with anti-phospho-c-Src^Tyr416^ antibody (see Methods). Note, relative to untreated cells, association of Gβ and c-Src^Tyr416^ proteins was significantly increased in IL-13-treated HASM cells; and formation of this protein complex was suppressed in IL-13-exposed cells that were pretreated with either anti-Gβγ peptide (20 µM) or gallein (10 µM), and also suppressed in IL-13-exposed HASM cells that were transfected with adeno-βARK-ct. The immunoblots shown in **A**–**C** are representative from 3–4 experiments.

Since signaling initiated by Gi-βγ was found to mediate ERK1/2-dependent PDE4-induced proasthmatic changes in responsiveness in β2AR-desensitized rabbit ASM tissues and cultured human ASM (HASM) cells [10], [11], [23], a series of studies were pursued to systematically evaluate the role of this signaling mechanism in regulating ASM function under the present conditions of ASM sensitization. We initially examined whether HASM cells treated with IL-13 exhibit Gβγ-regulated c-Src and ERK1/2 activation, and whether this phenomenon is attributed to direct protein-protein interaction between activated Gβγ and c-Src. Accordingly, the effects of Gβγ inhibition on IL-13-induced phosphorylation of c-Src protein at residue Tyr416, which denotes the activated autophosphorylated state of the kinase [37], and ERK1/2 were assessed using phospho-c-Src^Tyr416^- and phospho-ERK1/2-specific antibodies. Corresponding total levels of c-Src and ERK1/2 were also determined in the same immunoblot preparations by stripping the membranes and reincubating with anti-c-Src and -ERK1/2 antibodies. Treatment of HASM cells with IL-13 (50 ng/ml) elicited temporal increases in both c-Src^Tyr416^ and ERK1/2 phosphorylation that peaked at 10 and 30 min, respectively (Fig. 7A). As demonstrated in Fig. 7B, contrasting the lack of effect of pretreatment with the inert MPS peptide alone (20 µM), peak IL-13-induced phosphorylation of both c-Src^Tyr416^ and ERK1/2 was suppressed in HASM cells pretreated with maximally effective concentrations of either anti-Gβγ blocking peptide (20 µM) or gallein (10 µM). Similar results were also obtained when comparing the effects of IL-13 on c-Src^Tyr416^ and ERK1/2 phosphorylation in HASM cells at 24 hr following transfection with either an adenovirus vector expressing *lacZ* (adeno-LacZ), serving as a negative control, or adeno-βARK-ct, which encodes the C-terminal domain of βARK1 that was shown to block Gβγ signaling [14], [15], both at a multiplicity of infection (MOI) of 100. Whereas cells transfected with adeno-LacZ exhibited IL-13-induced c-Src^Tyr416^ and ERK1/2 phosphorylation, this effect of IL-13 was prevented in cells transfected with adeno-βARK-ct (Fig. 7B).

In light of the above results implicating Gβγ as an upstream regulator of IL-13-induced c-Src activation, co-immunoprecipitation studies were then conducted to determine whether IL-13 evokes a direct interaction between activated Gβγ and phosphorylated c-Src. The immunoblots in Fig. 7C demonstrate that, relative to unstimulated cells, enhanced co-localization of phosphorylated c-Src^Tyr416^ with immunoprecipitated Gβ was detected in IL-13-treated HASM cells (Fig. 7C; top panel), and that this induced association was abrogated in IL-13-exposed HASM cells that were pretreated with either anti-Gβγ blocking peptide or gallein, as well as in IL-13-exposed cells that were transfected with adeno-βARK-ct. Of note, these results were obtained under similar conditions of loading of immunoprecipitated Gβ, as evidenced by the immunoblots using anti-Gβ antibody in Fig. 7C (bottom panel). Thus, together with the above observations, these data support the concept that IL-13 stimulates direct coupling of activated Gβγ to c-Src, and that this signaling event is associated with ERK1/2 activation in the cytokine-exposed ASM.

### Gβγ regulates IL-13-induced transcriptional upregulation of PDE4: Role in mediating altered ASM responsiveness

**Figure 8:**
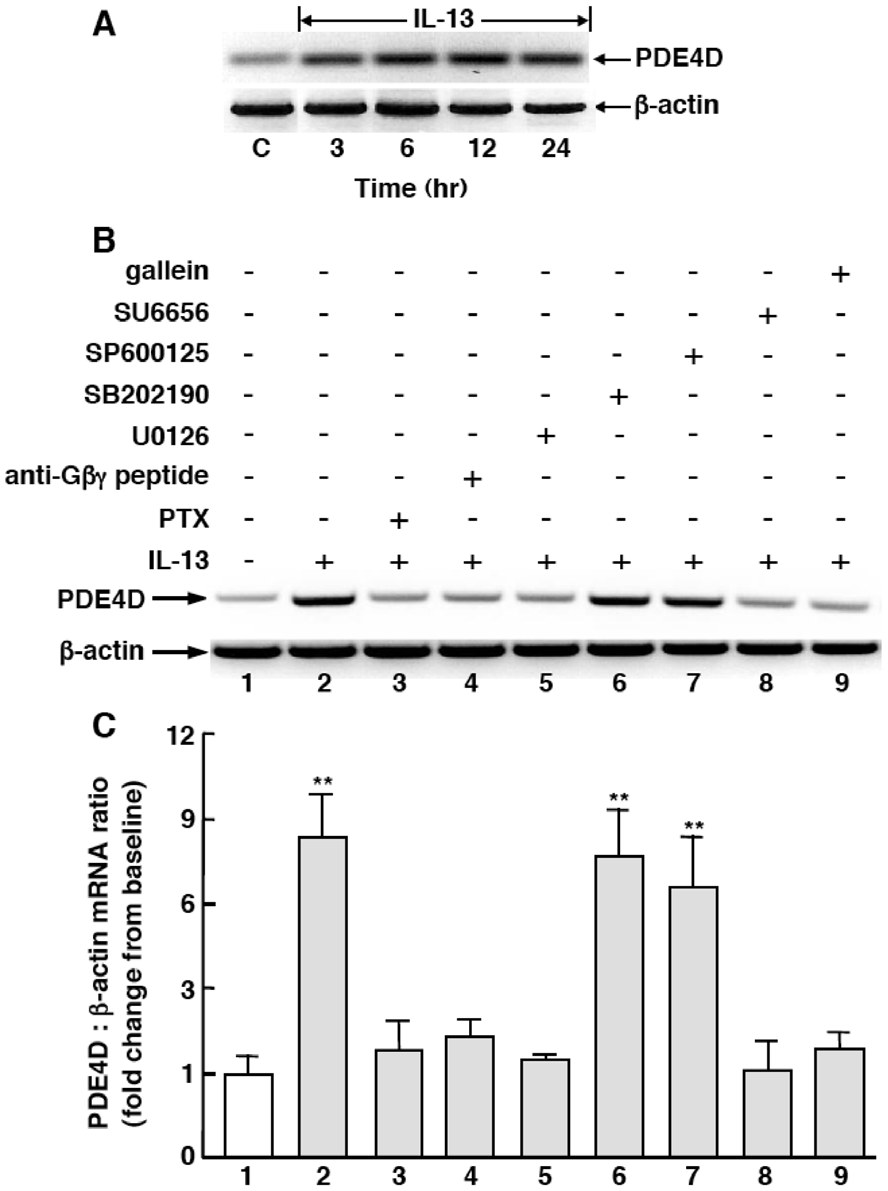
IL-13-induced Gi-βγ signaling mediates ERK1/2-dependent upregulation of PDE4D mRNA transcripts in HASM cells. (**A**) IL-13 elicits temporal increases in PDE4D mRNA expression in HASM cells, with peak induction of transcripts detected at 12 hr. A representative experiment (**B**), and corresponding densitometric analysis of PDE4D mRNA expression expressed as a ratio of β-actin (**C**), demonstrate that IL-13 induced PDE4D transcripts at 12 hr is abrogated in HASM cells that are pretreated either with PTX, anti-Gβγ blocking peptide, or inhibitors of either MEK-ERK1/2 (U0126), c-Src tyrosine kinase (SU6656) or gallein, whereas pretreatment with inhibitors of p38 MAPK (SB202190) or JNK (SP600125) has no effect. Data are mean ± SE values from 4 experiments (**p<0.01).

To further elucidate the mechanism by which Gβγ regulates the induction of PDE4 activity, we next investigated the effects of inhibition of Gβγ signaling and its suspected downstream effectors on IL-13-induced PDE4 expression in HASM cells. As shown in Fig. 8A, treatment of HASM cells with IL-13 (50 ng/ml×24 hr) evoked temporal increases in mRNA expression of *PDE4D*, the functionally dominant PDE4 subclass in HASM cells [38], [39], with peak expression detected at 12 hr and elevated levels sustained at 24 hr. As demonstrated by a representative experiment in Fig. 8B, and the corresponding results based on densitometric analysis of the data obtained in 4 such experiments in Fig. 8C, relative to control (vehicle-exposed) HASM cells (*lane 1*), IL-13-induced upregulation of *PDE4D* transcripts at 12 hr averaged ∼7.6-fold (*lane 2*), and this response was suppressed by pretreating IL-13-exposed cells with previously reported [10],[11],[23] maximally effective concentrations of either PTX (100 ng/ml; *lane 3*), which ADP ribosylates Gi protein, the anti-Gβγ blocking peptide (20 µM; *lane 4*), or the ERK1/2 inhibitor, U0126 (5 µM; *lane 5*). By comparison, induction of *PDE4D* transcripts by IL-13 was unaltered in cells pretreated with either the p38 MAPK inhibitor, SB202190 (10 µM; *lane 6*) or the JNK inhibitor, SP600125 (10 µM; *lane 7*), whereas the induction of *PDE4D5* transcripts was prevented in IL-13-exposed cells that were pretreated with either the c-Src family tyrosine kinase inhibitor, SU6656 (10 µM; *lane 8*) or gallein (10 µM, *lane 9*). Thus, consistent with earlier evidence demonstrating that activation of the Gβγ subunit of Gi protein elicits c-Src-induced downstream signaling via the Ras/c-Raf1/MEK-ERK1/2 pathway [14], [15], the above results support the notion that IL-13-induced PDE4 expression is regulated by Gi-βγ signaling coupled to c-Src activation that, in turn, leads to downstream ERK1/2-dependent induction of *PDE4D* transcripts.

**Figure 9:**
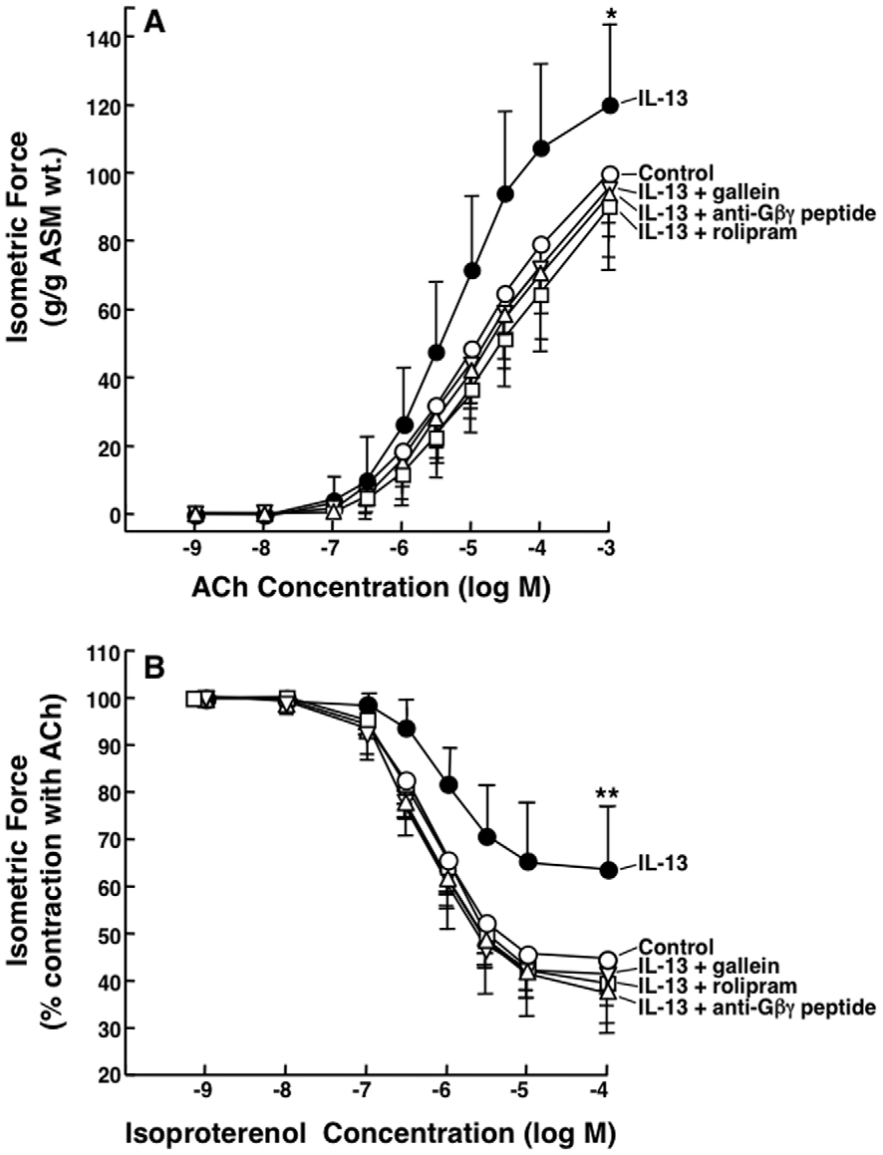
Inhibition Gβγ signaling prevents rolipram-sensitive changes in agonist responsiveness in IL-13-exposed rabbit ASM tissues. Relative to controls, ASM tissues sensitized with IL-13 (50 ng/ml×24 hr) exhibit significantly increased contractility to ACh (**A**) and impaired relaxation to isoproterenol (**B**). Inhibition of Gβγ signaling by pretreatment with anti-Gβγ blocking peptide or gallein, or inhibition of PDE4 activity with rolipram, prevents IL-13-induced changes in ASM responsiveness. Data are mean ± SD values from 5–6 experiments. ANOVA used for multiple comparisons of mean Tmax and Rmax values. *p<0.05; **p<0.01.

In concert with the above results, extended studies conducted in isolated rabbit ASM tissues demonstrated that (Fig. 9): 1) as previously described [34], relative to controls, ASM tissues exposed to IL-13 (50 ng/ml×24 hr) exhibit significantly heightened constrictor responses to ACh and impaired relaxation responses to isoproterenol; 2) as in atopic serum-sensitized tissues (Fig. 1), these IL-13-induced changes in ASM responsiveness are also prevented by pretreating the tissues with either rolipram or the anti-Gβγ blocking peptide; and 3) comparable inhibition of the pro-asthmatic effects of IL-13 is also seen in tissues pretreated with gallein (10 µM). Along with the above results, these observations support the concept that activation of the Gβγ subunit of Gi protein, which elicits c-Src-induced downstream signaling via the Ras/c-Raf1/MEK-ERK1/2 pathway [14], [15], leads to PDE4 upregulation and its consequent induction of proasthmatic changes in constrictor and relaxation responsiveness in OVA serum- and IL-13-sensitized ASM.

## Discussion

Whereas activation of the α subunits of the G proteins, Gq and Gs, is identified as primarily responsible for mediating acute ASM contraction and relaxation, respectively, in response to various bronchoactive agents [1], [40], signaling initiated by PTX-sensitive Gi proteins was shown to play a critical role in mediating the heightened constrictor and impaired relaxation responses that typify ASM tissues sensitized under different proasthmatic conditions [7]–[11]. The latter studies presumed that the proasthmatic role of PTX-sensitive Gi protein activation was attributed to an observed increase in ASM expression of the Gαi2 and Gαi3 isoforms [7]–[9]; however, it was subsequently demonstrated that transgenic mice overexpressing Gαi2 or a peptide inhibitor of Gαi2 in ASM do not exhibit increased ASM contractility to MCh [41]. Instead, it is now generally recognized that most physiological processes inhibited by PTX are mediated by the βγ subunits of Gi protein, rather than the α subunit, and that most Gβγ-dependent signaling arises from Gi protein [17], [42]–[45]. Consistent with this concept, our present observations demonstrated that inhibition of Gβγ activation prevented ERK1/2-dependent rolipram-sensitive changes in constrictor and relaxation responsiveness in rabbit ASM tissues sensitized with OVA serum or IL-13 (Figs. 1 and 9, respectively), and that this effect of Gβγ inhibition was associated with suppression of the upregulated PDE4 activity detected in these sensitized tissues (Fig. 6). Moreover, inhibition of Gβγ, resulting in its impaired direct activation of c-Src and accompanying ERK1/2 activation (Fig. 7), was also found to prevent PTX-sensitive induction of *PDE4D* mRNA transcripts by IL-13 in HASM cells (Fig. 8). Finally, in concert with the well documented causal relationship between PTX-sensitive Gβγ signaling and inflammation [17]–[22], the present results demonstrated that, together with abrogating *in vivo* airway hyperreactivity, inhibition of Gβγ signaling also suppressed the pulmonary inflammatory response to antigen challenge in OVA-sensitized rabbits (Figs. 2 and 3, respectively), and that these protective effects of Gβγ inhibition were associated with suppression of the upregulated PDE4 activity detected in the lungs of the OVA-challenged allergic rabbits (Fig. 6A). To our knowledge, these findings are the first to identify that Gβγ signaling leading to c-Src-induced ERK1/2-dependent upregulation of PDE4 activity plays a decisive role in regulating the altered airway function associated with the allergic asthmatic phenotype.

The present observations raise certain relevant considerations regarding potential mechanisms by which Gβγ signaling regulates the proasthmatic state. In this regard, our results generally agree with the substantial body of literature reporting that PTX-sensitive Gβγ signaling initiates critical proinflammatory events in various cell types [17]–[22], including those implicated in the pathobiology of allergic asthma. Accordingly, Gβγ-induced activation of the signaling molecules, PI3K and PLCβ2/β3, was shown to be an essential mechanism that regulates IgE-mediated degranulation by stimulated mast cells [18], [44], [45], as well as the recruitment and migration of leukocytes [17], [27], [46]. Moreover, it is noteworthy that PI3K and PLCβ2/β3 are also key regulators of ASM proliferation and contractility, respectively [1], [6], [40], and that the latter regulatory action may account, at least in part, for the reported PTX-sensitive increase in ASM contractility associated with heightened PLC-mediated inositol phosholipid signaling in ASM isolated from allergic rabbits [47], as well as in rabbit ASM passively sensitized with atopic asthmatic serum [5], [48]. Another mechanism relates to Gi-βγ-initiated activation of MAPK signaling, particularly that attributed to stimulation by Gi-βγ of c-Src-induced signaling via the Ras/Raf/ERK1/2 pathway [13]–[15]. The present observations provide several lines of evidence that implicate the latter Gi-βγ-regulated mechanism in mediating our observed proasthmatic changes in ASM contractility, as given by the results demonstrating key intermediate regulatory roles for G_i_-βγ, c-Src and ERK1/2 activation in mediating the upregulated PDE4 activity associated with altered agonist responsiveness in the sensitized ASM tissues, and induction of *PDE4D* transcripts by IL-13 in HASM cells. Furthermore, our extended observations demonstrated that activation of both c-Src and ERK1/2 by IL-13 in HASM cells was suppressed by pretreatment with different inhibitors of Gβγ and, based on related co-immunoprecipitation studies, c-Src tyrosine kinase was identified as a direct effector of Gβγ signaling (Fig. 7). Collectively, these data strongly implicate Gi-βγ signaling leading to c-Src-induced ERK1/2 activation and its upregulation of PDE4 expression as a key signaling mechanism that underlies the changes in airway function observed under the *in vivo* and *in vitro* experimental conditions used herein to simulate the allergic asthmatic state. Of significance, we previously demonstrated that the same Gi-βγ-driven mechanism coupled to stimulation of the c-Src/Ras/Raf/MEK-ERK1/2 pathway is also responsible for mediating the PDE4-induced proasthmatic changes in ASM contractility observed following prolonged heterologous or homologous β2AR-desensitization [10], [11]. Given this concurring evidence, along with a collection of earlier findings that the proasthmatic changes in ASM responsiveness evoked by either passive sensitization with human atopic asthmatic serum or exposure to proinflammatory cytokines or rhinovirus are also abrogated by pretreatment with PTX [7]–[9], the compelling consideration is raised that signaling via the Gβγ subunits of Gi protein plays a pivotal role in regulating the induction of altered ASM responsiveness under a variety of proasthmatic sensitizing conditions. This provocative consideration is worthy of future systematic investigation.

Cell-permeable peptides coupled to various cargo molecules have been used in animals models to successfully target specific intracellular signaling pathways implicated in disease, including in murine models of allergic airway disease [49]. Our rationale for using a cell permeable anti-Gβγ blocking peptide, comprised of an inert carrier MPS conjugated to the C-terminal thioredoxin-like domain of PhLP [17], is based on previous evidence demonstrating that: 1) PhLP is endogenously expressed in a wide variety of tissues, including lung [50], and acts as an ubiquitous inhibitor of Gβγ signaling by sequestering [50]–[53] or producing a conformational change in Gβγ protein [53]; 2) the MPS-conjugated anti-Gβγ blocking peptide is a highly effective inhibitor of α2-adrenergic receptor-mediated activation of MAPK [17]; and 3) in concert with the latter finding, we previously demonstrated that the anti-Gβγ blocking peptide abrogates PTX-sensitive ERK1/2 activation and its consequent induction of PDE4-mediated changes in responsiveness in β2AR-desensitized ASM, whereas comparable treatment with a MPS-conjugated anti-G_i_α3 peptide has no effect [11]. Consistent with this evidence, our present observations demonstrated that, together with its protective effects in OVA serum- and IL-13-sensitized isolated ASM tissues (Figs. 1 and 9, respectively), pretreatment with aerosolized anti-Gβγ blocking peptide prevented *in vivo* AHR evoked by antigen challenge in OVA-sensitized rabbits (Fig. 2). Moreover, pretreatment with anti-Gβγ blocking peptide also suppressed the pulmonary inflammatory response and reversed the predominance of IL-13 vs. IFN-γ cytokine levels in the lungs of the OVA-sensitized+challenged rabbits (Figs. 3 and 5). Of significance, these observations, as well as those identifying that the Gβγ signaling mechanism activated in the sensitized ASM involves ERK1/2-dependent upregulation of PDE4 activity, concur with evidence provided in earlier *in vivo* studies demonstrating that: 1) inhibition of ERK1/2 activation suppresses both the *in vivo* AHR and pulmonary inflammation elicited by antigen challenge in OVA-sensitized mice [54], [55]; and 2) increased PDE4 activity plays a decisive role in mediating both the heightened bronchoconstrictor responsiveness and airway inflammation evoked by allergen challenge in asthmatic subjects [28] and in animal models of allergic asthma [29]–[33]. Thus, when considered in light of the extended mechanistic observations described herein, the circumstantial evidence provided by the latter findings further substantiate the concept that Gβγ signaling associated with ERK1/2-dependent induction of PDE4 activity is importantly involved in mediating both the altered airway responsiveness and inflammation exhibited in the allergic asthmatic lung. In this context, it is important to indicate that the critical role identified herein for Gβγ signaling is based on evidence demonstrating the bronchoprotective action of anti-Gβγ treatment in preventing induction of the airway asthmatic response, and it remains to be determined whether this treatment is also effective in reversing the asthmatic response once manifested.

In further considering the present observations, it should be noted that, like pretreatment with the anti-Gβγ blocking peptide, the induction of *in vivo* AHR and altered agonist responsiveness in the isolated sensitized ASM tissues were also abrogated by pretreatment with gallein, a small molecule inhibitor of Gβγ signaling [17], [26], [27]. Unlike the anti-Gβγ blocking peptide, however, gallein did not suppress the pulmonary inflammatory response in OVA-sensitized+challenged rabbits (Fig. 3). While the disparity between these inhibitors with respect to their anti-inflammatory actions is not readily explained, one possibility relates to potential differences in their pharmacodynamic or pharmacokinetic properties that might influence their effects on the effector systems that regulate AHR and inflammation. Another reasonable explanation relates to potential differences in their mechanisms of Gβγ inhibition under the present experimental conditions. In this regard, it has been proposed that, among the small molecule inhibitors of Gβγ signaling examined to date, each has its distinctive spacial orientation of binding to the bioactive site (“hot spot”) on the Gβγ surface, thereby accounting for inhibition of only specific Gβγ-targeted effector interactions [17]. Accordingly, in extending this concept to the present observations, it is conceivable that gallein allows only for inhibition of those Gβγ-targeted effector interactions that regulate airway responsiveness but not those that mediate allergic lung inflammation, although it should be noted that gallein was found to inhibit carrageenan-induced footpad inflammation in mice [27]. On the other hand, by sequestering the Gβγ subunit, the anti-Gβγ blocking peptide is arguably capable of relatively greater inhibition of Gβγ interactions with different effector targets, including those that mediate both changes in airway responsiveness and inflammation. This interesting possibility remains to be systematically investigated.

In evaluating the implications of the present study, it must be emphasized that our observations pertain to studies conducted in an *in vivo* rabbit model of allergic airway disease and in isolated sensitized rabbit ASM tissues. Therefore, the extent to which these observations relate to the human condition is open to speculation. In this respect, it is noteworthy that our results are consistent with recent evidence that implicates upregulated PDE4 activity in mediating the airway responses to allergen challenge in asthmatic individuals [28], and the induction of altered HASM function [10], [11], [23], [56], [57]. Additionally, our data generated in the rabbit ASM tissues are consistent with those obtained in the cultured HASM cells, as both these experimental preparations exhibited complementary Gβγ-dependent changes in ASM function. These included compatible effects of specific inhibitors on the Gβγ-regulated signaling events mediating IL-13-induced PDE4 expression in HASM cells (Figs. 7 and 8) and altered responsiveness in rabbit ASM tissues (Fig. 9). Moreover, our findings regarding the Gβγ-regulated mechanism underlying the effects of IL-13 on ASM responsiveness are consistent with those in previous reports demonstrating that IL-13 evokes downstream ERK1/2-dependent changes in HASM contractility [58] and intracellular calcium mobilization [59], as well as pulmonary inflammation [60]. Regarding the latter, it should be noted that our *in vivo* observations in rabbits,concur with those in previous reports that also demonstrated a predominant neutrophilic pulmonary inflammatory response initially (i.e., up to 24 hours) following antigen challenge in sensitized mice [61]–[63] and rabbits [64], as well as the reported predominant neutrophilic inflammatory response detected in BAL samples obtained hours following airway antigen challenge in allergic asthmatic individuals [65]. In this regard, however, it is important to note that, given the frequent observance of eosinophilic infiltration at 24 hours after allergen challenge in other studies in OVA-sensitized mice, consideration must be given to other factors that may also contribute to our observed neutrophilic response, including LPS contamination of the administered OVA preparation. Finally, another relevant issue is that our observed changes in constrictor and relaxant responsiveness in the sensitized rabbit ASM tissues mimicked the perturbations in airway function that characterize the human asthmatic ASM phenotype, including enhanced constrictor responsiveness to cholinergic stimulation and impaired β2AR-mediated airway relaxation [66], [67]. Thus, in view of these considerations, we believe that the findings of the present study are applicable, at least in part, to the human condition.

In conclusion, this study is the first to report that Gβγ signaling associated with ERK1/2 activation and upregulated PDE4 activity plays a critical role in mediating the induction of airway hyperresponsiveness and inflammation in a rabbit model of allergic asthma. By identifying that Gβγ signaling mediates these key characteristic features of the airway asthmatic phenotype, the present findings support the consideration that future interventions targeted at modulating Gβγ function may yield new approaches to treat allergic airway disease.

## Methods

### Materials

All chemicals and reagents were purchased from Sigma-Aldrich unless otherwise indicated. Human ASM (HASM) cells were obtained from Bio Whittaker, Inc.

### Animals

Thirty-five young adult (6–8 months of age) male New Zealand White rabbits purchased from Covance were used in this study, which was approved by the Biosafety and Animal Research Committee of the Research Institute at Children's Hospital of Philadelphia.

### OVA sensitization and challenge

Rabbits were actively immunized with weekly intraperitoneal (i.p.) injections of 1 ml of an OVA-containing emulsion, comprising 2.5 mg OVA prepared from lyophilized powder (Grade V; ≥98% purity) in 1 part physiological saline and 1 part of Alum adjuvant (Pierce), for a total of 4 injections. One week later, similar to the approach previous described in OVA-sensitized rabbits [68], the animals received a single inhalation challenge of an aerosolized 7% solution of OVA in physiological saline, delivered at a flow rate of 8 l/min over ∼20 min via a compressor nebulizer (DeVilbiss, PulmoMate) connected to an oro-pharyngeal tube. The OVA challenges were conducted in the absence and presence of pre-nebulization at 1 hr prior to antigen challenge either with 0.5 mg/Kg of the inhaled glucocorticosteroid, budesonide (Pulmicort Respules; Astra-Zeneca), 1 mg/Kg of a cell permeable anti-Gβγ blocking peptide (AnaSpec), comprised of a membrane permeable sequence (MPS; 15 amino acids) conjugated to the Gβγ-sequestering C-terminal domain (28 amino acids) of phosducin-like protein [25], or 1 mg/Kg of the inert MPS peptide alone serving as a negative-control. The blocking Gβγ peptide and control MPS aerosolized solutions were prepared in 3 ml of physiological saline and nebulized over 15–20 min. In separate studies, at 1 hr prior to OVA challenge, control and OVA-sensitized rabbits were pretreated with an i.p. bolus injection of either vehicle alone (PBS) or 30 mg/Kg of gallein [26], a recently described small molecule inhibitor of Gβγ signaling [27] (Acros Organics).

### Measurement of *in vivo* bronchoconstrictor responsiveness

At 24 hr following OVA challenge, under initial general anesthesia with intramuscular injections of xylazine (10 mg/Kg) and ketamine (50 mg/Kg), rabbits were tracheotomized, paralyzed with pancuronium bromide (0.05 mg/Kg), and mechanically ventilated via an intra-tracheal cannula. The cannula was connected to a pneumotachograph to monitor airflow and a pressure transducer to record transthoracic pressure. As previously described [69], the flow and pressure signals were digitized and analyzed to determine breath-to-breath changes in respiratory system resistance (Rrs) and dynamic compliance (Cdyn) elicited by i.v. bolus injections of cumulatively increasing doses of MCh (0.001–0.15 mg/Kg).

### Lung histology and BALF analysis of inflammation

Following assessment of MCh responsiveness, the animals were sacrificed with an overdose of sodium pentobarbital (100 mg/Kg) and, similar to the approach previously described in OVA-senstized rabbits [68] the lungs were lavaged *in situ* with a total of 25 ml of normal saline, delivered by slowly injecting and aspirating five 5-ml aliquots via a pliable plastic catheter inserted into the intra-tracheal tube. The lungs were then excised and fixed in 10% formalin under a constant pressure of 20 cmH_2_0. The BALF return averaged between 67 and 79% of the total instilled volume. Total and differential cell counts in the BALF samples were assessed in Wright-stained cytospin preparations, with at least 500 cells counted per cytospin preparation. The levels of IL-13 and IFN-γ in the BALF supernatants were determined using ELISA kits (R&D Systems). Paraffin-embedded 4 µM sections of lung tissue obtained from the lower lobes of both formalin-fixed lungs were stained with hematoxylin and eosin (H&E) and examined in a blinded manner to assess inflammation.

### Preparation and *ex vivo* sensitization of rabbit ASM tissues

In separate experiments, naïve NZW rabbits were sacrificed with sodium pentobarbital (100 mg/Kg) and the tracheae were excised, cleaned of loose connective tissue, and the epithelium was removed by gently scraping with a cotton-tipped applicator, as previously described [7], [24]. The isolated tracheae were divided into equal ring segments, and each alternate segment was incubated overnight either with vehicle alone or serum isolated from control (non-sensitized) or OVA-sensitized rabbits at 24 hr following OVA inhalation challenge, or with IL-13 (50 ng/ml), both in the absence and presence of pretreatment with the anti-Gβγ blocking peptide (20 µM), MPS peptide alone (20 µM), gallein (10 µM), the ERK1/2 inhibitor, U0126 (5 µM), or the PDE4 inhibitor rolipram (10 µM). The tissues were exposed to each of these treatments for ∼3 hr prior to incubation with the sera or IL-13 preparations, and the treatments were maintained throughout the ensuing overnight period (∼18 hr) leading to the following pharmacodynamic studies.

### Pharmacodynamic studies of ASM tissue responsiveness

Following incubation under the different treatment conditions, the ASM tissue segments were placed in organ baths containing the same concentrations of their respective pharmacological treatments in modified Krebs-Ringer solution aerated with 5% CO2 in O2 and attached to force transducers to monitor isometric tension. As previously described [7]–[10], cholinergic contractility to cumulatively administered acetylcholine (ACh; 10^−9^ to 10^−3^ mol/L) was assessed and, after rinsing with fresh buffer, relaxation dose-response curves to isoproterenol (10^−9^ to 10^−4^ mol/L) were generated following initial half-maximal contraction of the tissues with ACh. The constrictor and relaxation dose-response curves were analyzed with respect to each tissue's maximal isometric contractile force (Tmax) to acetylcholine and maximal relaxation response (Rmax) to isoproterenol.

### Assay of cAMP PDE activity

Levels of total cAMP PDE activity were determined, as previously described [10], [11], using a colorimetric, non-radioactive enzymatic assay (Biomol), in control and passively sensitized ASM tissues, and in lung tissue sections isolated from the control and OVA-sensitized rabbits under the different treatment conditions. Following the above treatments, ASM and lung tissues sections were immediately frozen and then stored at −80°C. At the subsequent time of analysis, the tissue samples were thawed, then finely minced and homogenized in ice-cold 30 mM *N*-2-hydroxyethylpiperazine-*N*′-ethane sulfonic acid (pH 7.4) containing 0.1% Trion X-100, and PDE activity was standardized to protein content in the tissue samples [10], [11].

### Culture and treatment of ASM cells

HASM cells were grown in smooth muscle basal medium (SmBm) supplemented with 10% FBS (BioWhittaker) and maintained throughout in a humidified incubator containing 5% CO_2_ in air at 37°C. The experimental protocols involved growing the cells to ∼95% confluence in the above medium. Thereafter, in separate experiments, the cells were starved in unsupplemented Ham's F12 media for 24 hr, treated with different concentrations and for varying durations with IL-13, and then examined for induced changes in c-Src and ERK1/2 phosphorylation and PDE4D mRNA expression in the absence and presence of specific inhibitors, as described.

### Transfection of ASM cells with adeno-βARK-ct

Adenovirus (adeno)-βARK-ct, an adenovirus vector encoding the βARK1 carboxyl-terminal domain which blocks Gβγ signaling [14], [15], and adeno-β-gal, an adenovirus vector expressing *lacZ* as a negative control, were constructed using the AdenoX adenovirus construction kit (BD-Clontech). Recombinant plaques were isolated and propagated in HEK293 cells (Invitrogen), with viral purification using the cesium chloride gradient method, and viral titer detected by plaque assay. HASM cells were transfected with either of the adenoviral vectors at a MOI of 100, and experiments were conducted at 24 hr following the adenoviral transfections.

### Immunoblot analysis of c-Src and ERK1/2 phosphorylation

Levels of c-Src and ERK1/2 proteins, as well as phosphorylated c-Src at residue Tyr416 and ERK1/2 proteins, were detected by Western blot analysis of lysates isolated from HASM cells before and at various times after treatment with IL-13 in the absence and presence of specific inhibitors, as described. Following protein extraction and the addition of gel loading buffer, the extracts were loaded on a 10% SDS-PAGE gel for immunoblotting after transfer to a PVDF membrane. The membranes were then incubated overnight with monoclonal mouse anti-human primary antibodies directed against c-Src, phospho-c-Src^Tyr416^, ERK and phospho-ERK1/2 (Cell Signaling Technology), and levels were detected by ECL after a 1-hr incubation with a 1∶2,000 dilution of HRP-conjugated secondary antibody, followed by exposure to autoradiography film. The protein band intensities were quantified by densitometry.

### Co-immunoprecipitation studies

Untreated and IL-13-treated HASM cells were prepared for co-immunoprecipitation studies under native conditions in order to preserve protein-protein associations. After indicated treatment, cells were harvested and then lysed with lysis buffer. 1.5 mg of Protein G Dynabeads (Invitrogen) were incubated with 10 µg of anti-Gβ rabbit IgG (Millipore) and incubated for 10 min at room temperature with rotation. Following several washes, the bound bead/antibody complex was added to sample, mixed by pipetting, and incubated for 2 hr at 4°C with rotation. The captured bead/Protein G/antigen complex was then washed several times and eluted at pH 3.0 with rotation at room temperature. The precipitated immunocomplexes were subsequently analyzed by immunoblotting using anti-phospho-c-Src^Tyr416^ antibody.

### Expression of PDE4D mRNA transcripts

After attaining confluence, the cells were starved in unsupplemented Ham's F12 media for 24 hr, subsequently treated with IL-13, and then examined under different experimental conditions for induced changes in PDE4D mRNA expression, determined by RT-PCR as previously described [10], [11], [23].

### Detection of PDE4D mRNA transcripts

Total RNA was extracted from untreated and IL-13-treated HASM cells, in the absence and presence of co-treatment with specific inhibitors, using the TRIzol method (Invitrogen). cDNAs were then isolated by RT-PCR using the SuperScript First Strand Synthesis System kit from Invitrogen, with the following oligonucleotide primer sets (Integrated DNA Technologies): for PDE4D, 5′-CGGAGATGACTTGATTGTGAC-3′ (forward) and 5′-CGTTCCTGAAAAATGGTGTGC-3′ (reverse); and for β-actin, 5′-GAGAAGAGCTACGAGCTGCCTGAC-3′ (forward) and 5′-CGGAGTACTTGCGCTCAGGAGGAG-3′ (reverse). The reaction volume was 20 µl and cycling conditions used were 35 cycles of 30 sec denaturation at 95°C, followed by 30 sec annealing at 60°C and elongation at 72°C for 30 sec. Ex-Tag (Takara Biotechnology) was used as DNA polymerase.

### Statistical analyses

Results are expressed as mean ± SE values. Comparisons between groups were made using the Student t test (2-tailed), ANOVA with Tukey posttest analysis, and the nonparametric Kruskal-Wallis test with Dunn's posttest analysis, where appropriate. A probability of <0.05 was considered statistically significant. Statistical analyses were conducted by using the Prism computer program by Graph Pad Software Inc.

## Supporting Information

Figure S1 **Inhibition of Gβγ signaling prevents induced changes in agonist responsiveness in OVA serum-sensitized rabbit ASM tissues.** Relative to untreated (vehicle-exposed) controls, ASM tissues exposed to control serum exhibit similar Tmax responses to ACh (**A**) and Rmax responses to isoproterenol (**B**) both in the absence and presence of pre-treatment with either MPS peptide alone or anti-Gβγ blocking peptide. By comparison, OVA serum-exposed ASM tissues exhibit significantly increased Tmax responses (**A**) and reduced Rmax responses (**B**) that are prevented by pre-treatment with anti-Gβγ blocking peptide, whereas pre-treatment with MPS alone has no effect. Data are mean ± SD values from 4–7 experiments. Treated tissues are compared to untreated (vehicle-exposed) controls using unpaired two-tailed Student t-test. *p<0.05; **p<0.01.
(TIF)

Figure S2 **Anti-Gβγ blocking peptide prevents** ***in vivo*** **antigen-induced airway hyperresponsiveness in OVA-sensitized rabbits.** Relative to OVA-challenged control (non-sensitized; n = 4) rabbits, MCH-induced decreases in Cdyn are significantly enhanced at 24 hr following antigen challenge in OVA-sensitized rabbits (n = 4). This heightened bronchoconstrictor responsiveness to MCh is suppressed in OVA-sensitized rabbits that are treated either with inhaled anti-Gβγ peptide (1 mg/Kg; n- = 4) or budesonide (0.5 mg/Kg; n = 3) prior to antigen challenge. Note: Data represent Cdyn responses associated with corresponding Rrs responses shown in Fig. 2. Data are mean ± SE values. ANOVA used for multiple comparisons of mean Rrs values. *p<0.05; **p<0.01.
(TIFF)
