# Supplementary figures and images for: G Protein βγ-Subunit Signaling Mediates Airway Hyperresponsiveness and Inflammation in Allergic Asthma
Source: PLoS One. 2012 Feb 22;7(2):e32078. doi: 10.1371/journal.pone.0032078 (PMC3284547; doi:10.1371/journal.pone.0032078)

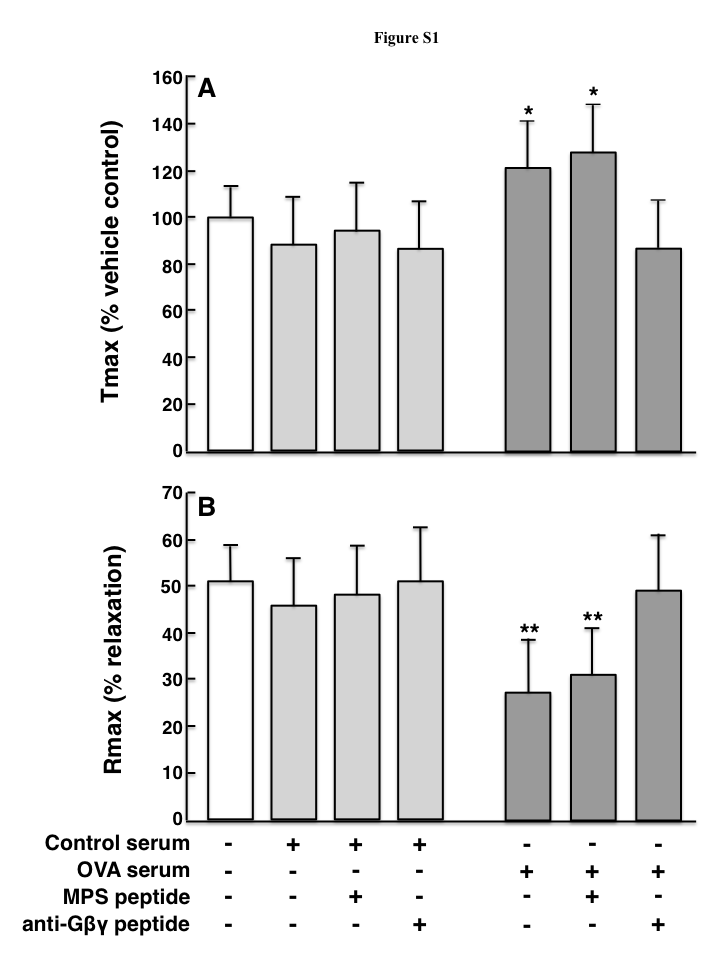

Supplement: Figure S1 — Inhibition of Gβγ signaling prevents induced changes in agonist responsiveness in OVA serum-sensitized rabbit ASM tissues. Relative to untreated (vehicle-exposed) controls, ASM tissues exposed to control serum exhibit similar Tmax responses to ACh (A) and Rmax responses to isoproterenol (B) both in the absence and presence of pre-treatment with either MPS peptide alone or anti-Gβγ blocking peptide. By comparison, OVA serum-exposed ASM tissues exhibit significantly increased Tmax responses (A) and reduced Rmax responses (B) that are prevented by pre-treatment with anti-Gβγ blocking peptide, whereas pre-treatment with MPS alone has no effect. Data are mean ± SD values from 4–7 experiments. Treated tissues are compared to untreated (vehicle-exposed) controls using unpaired two-tailed Student t-test. *p<0.05; **p<0.01. (TIF) [file pone.0032078.s001.tif]

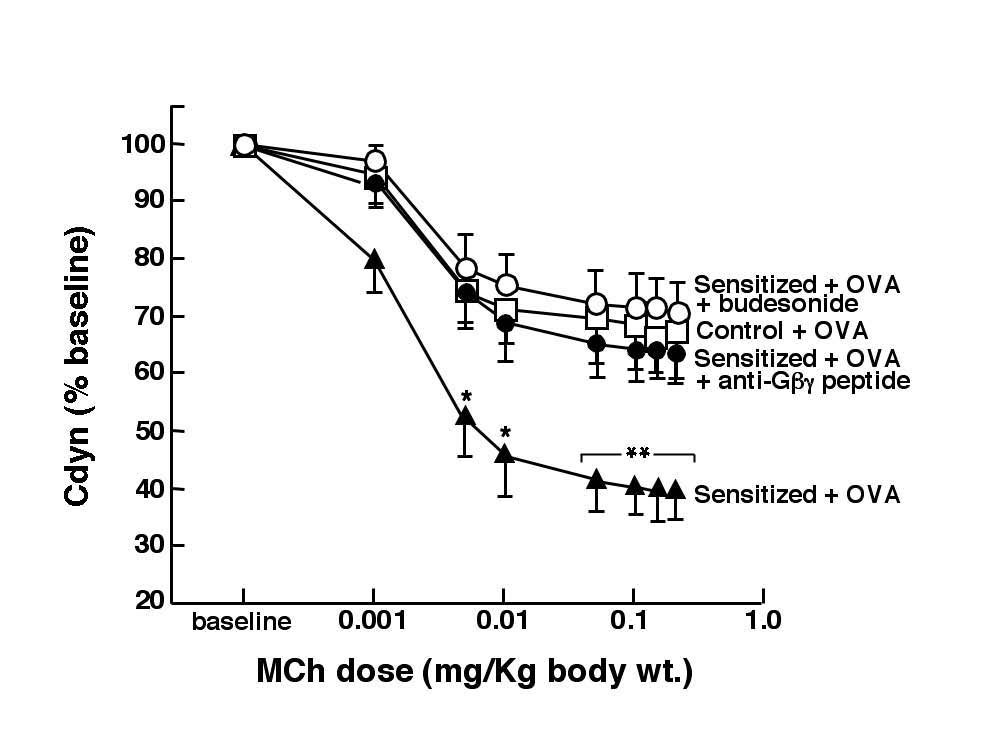

Supplement: Figure S2 — Anti-Gβγ blocking peptide prevents in vivo antigen-induced airway hyperresponsiveness in OVA-sensitized rabbits. Relative to OVA-challenged control (non-sensitized; n = 4) rabbits, MCH-induced decreases in Cdyn are significantly enhanced at 24 hr following antigen challenge in OVA-sensitized rabbits (n = 4). This heightened bronchoconstrictor responsiveness to MCh is suppressed in OVA-sensitized rabbits that are treated either with inhaled anti-Gβγ peptide (1 mg/Kg; n- = 4) or budesonide (0.5 mg/Kg; n = 3) prior to antigen challenge. Note: Data represent Cdyn responses associated with corresponding Rrs responses shown in Fig. 2. Data are mean ± SE values. ANOVA used for multiple comparisons of mean Rrs values. *p<0.05; **p<0.01. (TIFF) [file pone.0032078.s002.tiff]
